# Antimicrobial point prevalence surveys in two Ghanaian hospitals: opportunities for antimicrobial stewardship

**DOI:** 10.1093/jacamr/dlaa001

**Published:** 2020-02-18

**Authors:** Daniel Kwame Afriyie, Israel A Sefah, Jacqueline Sneddon, William Malcolm, Rachel McKinney, Lesley Cooper, Amanj Kurdi, Brian Godman, R Andrew Seaton

**Affiliations:** 1 Pharmacy Department, Ghana Police Hospital, Accra, Ghana; 2 Department of Pharmacy, Keta Municipal Hospital, Keta-Dzelukope, Volta Region, Ghana; 3 Scottish Antimicrobial Prescribing Group, Healthcare Improvement Scotland, Delta House, 50 West Nile Street, Glasgow G1 2NP, UK; 4 Health Protection Scotland, NHS National Services Scotland, Glasgow, UK; 5 NHS Lothian, Western General Hospital, Crewe Road South, Edinburgh, UK; 6 Strathclyde Institute of Pharmacy and Biomedical Sciences, Strathclyde University, Glasgow, UK; 7 Department of Pharmacology, College of Pharmacy, Hawler Medical University, Erbil, Iraq; 8 Division of Clinical Pharmacology, Karolinska Institute, Karolinska University Hospital Huddinge, Sweden; 9 School of Pharmacy, Sefako Makgatho Health Sciences University, Garankuwa, Pretoria, South Africa; 10 Queen Elizabeth University Hospital, Govan Road, Glasgow, UK; 11 University of Glasgow, Glasgow, UK

## Abstract

**Background:**

Improved knowledge regarding antimicrobial use in Ghana is needed to reduce antimicrobial resistance (AMR). This includes point prevalence studies (PPSs) in hospitals. Objectives were to: (i) provide baseline data in two hospitals [Keta Municipal Hospital (KMH) and Ghana Police Hospital (GPH)] and identify priorities for improvement; (ii) assess the feasibility of conducting PPSs; and (iii) compare results with other studies.

**Methods:**

Standard PPS design using the Global PPS paper forms, subsequently transferred to their template. Training undertaken by the Scottish team. Quality indicators included: rationale for use; stop/review dates; and guideline compliance.

**Results:**

Prevalence of antibiotic use was 65.0% in GPH and 82.0% in KMH. Penicillins and other β-lactam antibiotics were the most frequently prescribed in both hospitals, with third-generation cephalosporins mainly used in GPH. Antibiotic treatment was mainly empirical and commonly administered intravenously, duration was generally short with timely oral switching and infections were mainly community acquired. Encouragingly, there was good documentation of the indications for antibiotic use in both hospitals and 50.0%–66.7% guideline compliance (although for many indications no guideline existed). In addition, almost all prescribed antibiotics had stop dates and there were no missed doses. The duration of use for surgical prophylaxis was generally more than 1 day (69.0% in GPH and 77.0% in KMH).

**Conclusions:**

These two hospitals were the first in Ghana to use the Global PPS system. We found the PPS was feasible, relatively rapid and achieved with limited training. Targets for improvement identified included reduction of broad-spectrum antibiotics and duration of treatment.

## Introduction

Unnecessary use of antibiotics is a significant and modifiable driver for antimicrobial resistance (AMR), associated with increased morbidity and mortality^1^ as well as cost.^2–6^ AMR is of particular concern in low- and middle-income countries (LMICs), due to recent marked increases in antimicrobial utilization.^7–10^ Rising AMR rates have resulted in regional, national and global initiatives to improve future antibiotic use through implementation of antimicrobial stewardship programmes (ASPs),^11–19^ with variable implementation and outcomes in LMICs due to limited resources.^17^^,^^20–25^

Inappropriate use of antimicrobials in hospitals is widely reported,^21^^,^^26–35^ exacerbated by high rates of HIV, TB and malaria in sub-Saharan African countries,^21^^,^^36^ coupled with variable diagnostic resources. Lack of equipment and funding models for diagnostics leads to empirical antibiotic use without culture and susceptibility testing. To improve hospitals’ antimicrobial use, LMICs have developed national action plans (NAPs) with key elements of surveillance data,^12^^,^^21^^,^^28^ national standard treatment guidelines (NSTGs)^16^^,^^37^ and quality indicators focused on limiting the use of broad-spectrum agents.^38–41^ Awareness raising via Drugs and Therapeutics Committees (DTCs) and education of healthcare staff^17^^,^^34^^,^^42–46^ are particularly important as there is variable knowledge of AMR amongst clinicians and managers.^47–51^ A key target is reducing the use of WHO ‘Watch and Reserve’ category antibiotics^13^ to preserve their activity and promote recommended ‘Access’ category agents for the majority of infections.

In Ghana, understanding of antimicrobial utilization patterns is required to support recent policy intentions.^52–54^ The Ghanaian Ministry of Health has developed NSTGs, including those for the management of common infections, and has launched a 5 year NAP (2017–21).^55^^,^^56^ The NAP covers improving knowledge of AMR, establishing surveillance of antimicrobial consumption, optimizing antimicrobial use and supporting sustainable investment in AMR reduction.

Several studies in Ghana have evaluated the use of antimicrobials in hospitals, as well as physicians’ knowledge and attitudes towards AMR.^33^^,^^57–60^ These studies show a high prevalence of antibiotic use, long treatment durations and high use of broad-spectrum agents, coupled with a lack of awareness of AMR. Following a Fleming Fund AMR summit^61^ and utilizing the Commonwealth Partnerships for Antimicrobial Stewardship (CwPAMS),^62^ actions from the Ghanaian NAP^63^ will be progressed to accelerate development of ASPs. In the absence of continuous audit data or comprehensive surveillance data, point prevalence surveys (PPSs) are an established methodology to assess and monitor antimicrobial prescribing in hospitals.^30^^,^^31^^,^^64–66^ Consequently, an initial CwPAMS activity led by a team from the Scottish Antimicrobial Prescribing Group (SAPG) was to support a PPS in one small urban hospital and one small rural hospital using the Global PPS system (GPPSS).^22^^,^^67^ The objectives were to firstly provide baseline data for each hospital and identify priorities for improvement to support antimicrobial stewardship (AMS). Secondly, to assess the feasibility of PPSs using a standardized tool with respect to the manpower needed following concerns in other LMICs.^21^^,^^31^^,^^68^ Thirdly, to compare results with other hospitals in Ghana involved in the CwPAMS initiative, with other sub-Saharan African countries and beyond through the GPPSS. The findings will inform bespoke multiprofessional training aimed at changing behaviours amongst staff in each hospital and future quality improvement programmes as part of local ASPs.

## Methods

### Study design and settings

The PPS was conducted in May 2019 using the GPPSS methodology.^28^^,^^69^ The two hospitals were Keta Municipal Hospital (KMH), a 110 bed government hospital in the rural Volta district of Ghana, and the Ghana Police Hospital (GPH), a 100 bed hospital in the capital city, Accra. KMH has six wards serving adult and paediatric medical populations, gynaecological surgery and obstetrics and is staffed by 273 temporary and permanent staff. GPH provides healthcare for police officers and their families but also serves the local population.^58^^,^^70^ The case mix in GPH is similar to KMH except there are additional facilities for specialist surgery and orthopaedics. GPH has 175 core clinical staff.^70^

### Training and data collection

Training on using the GPPSS data collection forms was undertaken by J.S., R.A.S. and R.M., based on their experience with SAPG in Scotland.^71^^,^^72^ Three healthcare professionals (either a pharmacist, a pharmacist technician or a nurse) were trained in each hospital to facilitate working. This was done in pairs of one UK and one Ghanaian professional to collect the PPS data. Local ethics approval and data collection was coordinated by the local pharmacy leads (D.K.A. and I.A.S.).

The detailed GPPSS methodology is explained elsewhere.^28^ All inpatients that had stayed overnight and remained on the ward at 08:00 on the day of the survey were included. Data were collected only from those patients who were receiving at least one antimicrobial for treatment or prophylaxis (medical or surgical) at the time of the survey. Antimicrobials included were Anatomical Therapeutic Chemical (ATC) J codes (anti-infectives for systemic use): J01(antibacterials), J02 (antimycotics), J04 (antimycobacterials) and J05 (antivirals).^73^ Exclusion criteria included short-stay patients (not admitted as an inpatient), those discharged before 08:00 on the day of the survey and those attending outpatient specialist clinics.

Data were collected using GPPSS paper forms and transferred to the online GPPSS. Ward data included specialty, bed capacity and number of admitted patients. Patient data included age, gender, reason for antimicrobial prescribing, prescribed antimicrobials and dosage regimen, and causative microorganisms if available. Only information from the patients’ notes were included in the study. There was no contact with healthcare professional staff to clarify the clinical information collected to improve its accuracy as this could be a focus of future quality improvement programmes.

Surgical prophylaxis included any antimicrobial administered to prevent surgical-site infections (SSIs) and medical prophylaxis was defined as the use of antibiotics to prevent infections in patients with non-surgical conditions.^21^^,^^28^ Infections were considered as community-acquired infections (CAIs) if symptoms of infection were present on admission or appeared <48 h after admission and as healthcare-associated infection (HAI) if symptoms appeared 48 h or more after admission, based on review of patients’ notes.^28^^,^^31^^,^^33^

### Antimicrobial utilization and quality indicators

Antimicrobial utilization was stratified by ward type (adults and paediatrics, surgical, obstetric/gynaecological and medical), antimicrobial class—ATC classification,^73^ by CAI or HAI and by treatment or prophylaxis.

Antimicrobial utilization was assessed against agreed quality indicators based on the GPPSS and other studies.^21^^,^^31^^,^^32^^,^^68^^,^^74^^,^^75^ These included: indication for antimicrobial use documented in the patient notes; compliance with guidelines for documented indication (where guidance was available); stop or review date for antimicrobial use documented in the notes (and medicine chart); whether antimicrobial prescription was empiric or targeted based on an identified pathogen; and duration of surgical prophylaxis. The guidelines used were the seventh edition of the NSTG of Ghana.^76^

Antimicrobial utilization in the two hospitals was compared against each other, with other African countries in the Global PPS^28^ and with other hospitals in Ghana^67^^,^^68^ and sub-Saharan African countries (Botswana, Kenya, Nigeria and South Africa).^21^^,^^32^^,^^35^^,^^57^^,^^59^

### Ethics

Formal ethics approval was not required at either hospital as there was no direct patient contact and all data was anonymized. Local DTCs supported the surveys and the medical superintendent and local management team in each hospital granted clinical permission before commencement of the PPS.

## Results

Data were collected from prescription charts and patient notes in all wards over 2 h on a single day in each hospital. Review and discussion of the data and comparison of prescriptions against available guidelines took place between all data collectors over an additional 2 h in each hospital. The Lead Pharmacist at each hospital (D.K.A. and I.A.S.) entered data onto the GPPSS online site. In GPH, work to realign ward designations and address other issues with the GPPSS was required over 2 days then data entry took a further 7 h. In KMH, there was similar realignment of ward destinations followed by data entry, carried out during the course of 1 week.

A number of general observations not specified within the GPPSS methodology were noted separately. There was a low prevalence of known HIV-positive patients (one patient in each hospital) and only three patients (all in KMH) being treated for TB, no patients were observed with a record of penicillin allergy and no patients were being treated presumptively for infection with MRSA.

### Prevalence of antimicrobial use

The overall prevalence of antibiotic use was 65.0% in GPH and 82.0% in KMH. Prevalence rates ranged from 46.7% to 100.0%, depending on the clinical specialty and patient population (Table 1). Rates of antimicrobial utilization were similar in adult medical wards (57.1%, 55.6%) and in adult surgical wards (46.7%, 50.0%) in GPH and KMH, respectively. More paediatric patients in KMH received antimicrobials (100.0%) than in GPH (76.9%).

**Table 1.** Percentage of overall antimicrobial prevalence by hospital and type of ward

	Adult			Paediatric			
	total	AMW	ASW	total	PMW	PSW	NMW
GPH, % (*n*)^a^	57.1 (49)	73.7 (19)	46.7 (30)	76.9 (10)	77.8 (9)	100.0 (1)	100.0 (3)
KMH, % (*n*)^a^	55.6 (90)	56.6 (76)	50.0 (14)	100.0 (11)	100.0 (11)	0	0
Africa, Global PPS (%)^b^ (70 hospitals)	64.2	63.9	59.0	79.4	99.4	77.9	85.7

Adult total, overall antimicrobial prevalence in adult wards; AMW, adult medical ward; ASW, adult surgical ward; paediatric total, overall antimicrobial prevalence in wards admitting children; PMW, paediatric medical ward; PSW, paediatric surgical ward; NMW, neonatal medical ward.

a Results are shown as percentage antimicrobial prevalence (*n*, number of treated patients).

b Based on a total of 70 hospitals.

### Antimicrobial prescribing

**Figure 1.**
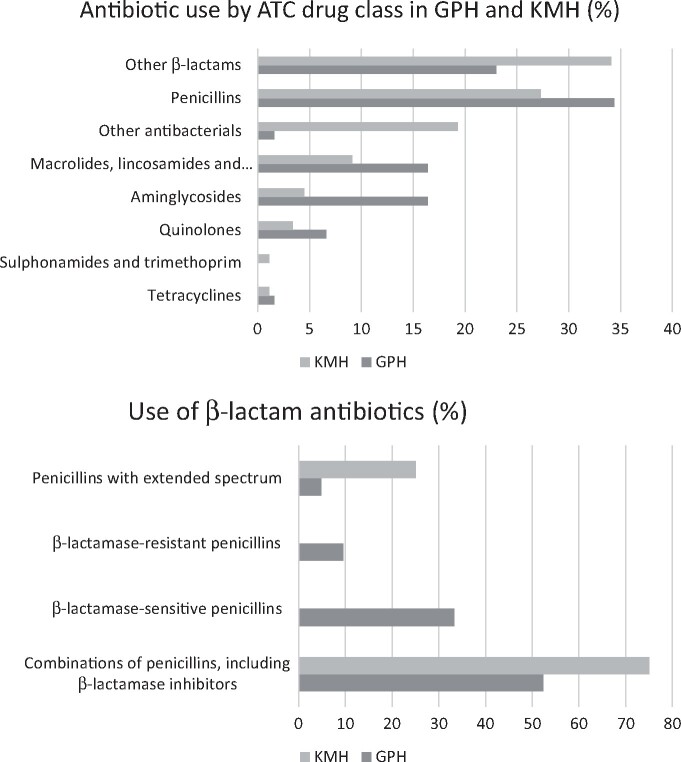
Details of antibiotics used.

Penicillins and other β-lactam antibiotics were the most frequently prescribed antibiotics in both hospitals, with combinations of penicillins with a β-lactam inhibitor (co-amoxiclav) the most commonly prescribed β-lactam antibiotic (Figure 1). Patterns of use of second- and third-generation cephalosporins varied between the two hospitals, with GPH using mainly third-generation agents (82.6%) while KPH used mainly second-generation agents (63.3%). Neither hospital had any prescriptions for carbapenems.

In both hospitals, antibiotics were commonly administered intravenously and the use of more than one antibiotic concurrently was also common (Table 2). Neonates were excluded from this data analysis as there were only three, all in GPH.

**Table 2.** Key prescription patterns across the two hospitals

	Percentage (*n*) with this characteristic	
Characteristic	GPH	KMH
IV therapy	68.6 (24)	55.9 (33)
Multiple antibiotic diagnosis	38.5 (15)	33.8 (22)
Multiple antibiotic patient	54.3 (19)	44.1 (26)

Multiple antibiotic diagnosis is defined as patients receiving >1 antibiotic for a single diagnosis. Multiple antibiotic patient is defined as receiving >1 antibiotic at the patient level.

### Indication for prescribing

Antimicrobials were predominantly used for CAI rather than HAI in GPH and solely for CAI in KMH. All but one prescription (at GPH) was empirical rather than targeted in both hospitals. Use of antibiotics for medical and surgical prophylaxis varied between the two hospitals (Table 3). The types of infections treated were similar across both hospitals but in KMH there were more non-severe respiratory tract infections: upper respiratory tract infections (URTIs); ear, nose and throat infections; and bronchitis.

**Table 3.** Summary of indications and specific diagnosis associated with antibiotic use

	Percentage (*n*) with characteristic	
Characteristic	GPH	KMH
Indication for antibiotic use		
CAI		
total	79.5 (35)	100 (80)
empirical	100 (35)	98.8 (79)
targeted	0	1.2 (1)
HAI		
total	20.5 (9)	0
empirical	100 (9)	0
targeted	0	0
prophylactic use		
medical	40.9 (9)	27.8 (5)
surgical	59.1 (13)	72.2 (13)
Top 11 infection diagnoses		
skin and soft tissue	26.9 (7)	11.5 (6)
obstetric/gynaecological infections	15.4 (4)	7.7 (4)
sepsis	15.4 (4)	11.5 (6)
ear, nose and throat infections		7.7 (4)
lower UTIs	11.5 (3)	3.8 (2)
pneumonia or LRTIs	11.5 (3)	25 (13)
gastrointestinal infections	7.8 (2)	13.5 (7)
upper UTIs	7.8 (2)	3.8 (2)
URTIs	3.8 (1)	
intra-abdominal sepsis		3.8 (2)
bronchitis		1.9 (1)

LRTI, lower respiratory tract infection.

### Antimicrobial quality indicators

There were some differences in the quality indicators between the two hospitals (Table 4) with overall good documentation of the indication for antibiotic administration in the patients’ notes in both hospitals. For many indications, guideline compliance could not be assessed as they were not included in the NSTGs. Where a guideline was available, compliance with the choice of agent was ≥50% in both hospitals for both medical and surgical patients.

**Table 4.** Key quality indicators for antimicrobial prescribing

	GPH		KMH		Africa Global PPS^a^	
Quality indicator	medical	surgical	medical	surgical	medical	surgical
Indication for antibiotic use recorded	100 (41)	85 (17)	88 (66)	84.5 (11)	60.8 (1839)	57.6 (1230)
Guidelines missing	46.3 (19)	70 (14)	1.3 (1)	46.2 (6)	24.1 (729)	43.9 (938)
Guideline compliant	62.5 (10)	66.7 (4)	55.4 (31)	50 (2)	55.9 (670)	61.2 (370)
Stop/review date in notes	92.7 (38)	95 (19)	98.7 (74)	100 (13)	29.1 (880)	32.4 (693)

Results are shown as percentage (*n*).

a Carried out in 70 hospitals.

No treatment was based on microbiology data in GPH and it was only used for one patient in KMH (2.2%). Culture and susceptibility testing is not covered by the national insurance scheme, so patients have to pay for these investigations.

Duration of surgical prophylaxis was typically more than 1 day, with no single-dose prophylaxis in either hospital. In 69.0% of patients in GPH, prophylaxis lasted >1 day and in 77.0% of patients in KMH, prophylaxis lasted >1 day.

## Discussion

Key findings included, firstly, that the PPS was feasible in both hospitals and was achieved with limited resources and minimal training of a multidisciplinary team including pharmacy technicians and nurses. In each hospital, six healthcare workers spent approximately 2 h on training and orientation, 4 h on data collection and validation then the lead pharmacist in each hospital spent a further 12–15 h checking and entering the data into the online system. It is likely that repeat PPSs will take less time as staff will be familiar with the process. It is envisaged that eventually direct electronic data collection may be considered, which will reduce data entry time further. This is important information given limited resources of time, available personnel and monies in LMICs. We acknowledge that both hospitals in Ghana had fewer inpatients than in the recently published PPS in Botswana, which took 3 to 5 days in their district hospitals using one or two data collectors and 10 days in a large referral hospital using two data collectors.^21^ In Pakistan, data from wards in each hospital were collected over 2 to 4 weeks.^31^ In South Africa, following considerable time taken with their previous paper-based PPS system, an App has been developed to speed up data collection and analysis.^68^ The GPPSS facilitates online real-time data entry but, for those new to PPSs, paper forms support learning and build confidence amongst data collectors. It is difficult to quantify the time required for a PPS as its feasibility depends on several factors including the maturity of ASPs, availability of staff with dedicated time for data collection and the ease of finding information in clinical systems. In the UK^64^^,^^65^ and across Europe, PPSs and ASPs are well established whereas in LMICs this journey is just beginning.

We acknowledge the limitations of a single PPS in that it cannot show trends and reasons for particular prescribing practices may reflect the particular staff behaviours on the day of data collection. However, this work is part of a larger project that will explore these factors. As some ward-based staff may have been aware that the team from Scotland were coming to support data collection it is possible this could have influenced prescribing practice.

The prevalence of antibiotic use (GPH 65.0%, KMH 82.0%) is similar to in two studies from two hospitals in Botswana (70.6%)^21^ and in Kenya (67.7%)^32^ but higher than in two studies from two hospitals in South Africa (31.0% and 38.5%).^67^^,^^68^ This compares with 50.0% for the 12 participating hospitals from five African countries in the 2015 GPPSS study.^28^ There were low rates of HIV and TB in the two hospitals in Ghana compared with Botswana, where at least 40% of inpatients in their PPS had HIV and over 25% had TB.^21^ The comparatively high prevalence of antibiotic use in GPH and KMH will be explored further during forthcoming education and quality improvement sessions focused on developing ASPs.

There appeared to be fewer antibiotic options available in KMH versus GPH, which may reflect availability and supply issues in more remote rural settings reported in other LMIC countries.^77–80^ This may lead to a greater reliance on broader-spectrum antibiotics, as seen in the differing use of β-lactam/β-lactam inhibitor products between the two sites. Both hospitals had significant use of cephalosporins (GPH 24.0% and KMH 27.0% of total antibiotics used). GPH used more third-generation cephalosporins while KMH used more from the second generation. All except first-generation cephalosporins are in the WHO ‘Watch’ category of antibiotics so use should be limited.^81^^,^^82^ There was low use of fluoroquinolones in both hospitals (6.6% in GPH and 3.4% in KMH), which is similar to the African hospitals in the GPPSS study and Botswana.^21^^,^^28^ The promotion of narrower-spectrum antibiotics, particularly for respiratory tract infections and urinary tract infections (UTIs), is a key focus for global stewardship by the WHO in their AwaRe classification within the Essential Medicines List.^81^^,^^82^ Promotion of the use of WHO ‘Access’ category antibiotics for these infections is key for ASPs to limit the use of broad-spectrum agents with their associated potential for driving AMR.

A key performance indicator for the CwPAMS work is the proportion of broad-spectrum antibiotics based on the GPPSS categorization. The proportion was similar in both hospitals (GPH 46.0%, KMH 46.6%) and largely due to high cephalosporin use.

It was also encouraging to see good documentation of indications for antibiotic use in both hospitals (Table 4) and recording of the duration of antibiotic treatment in the notes and medicine chart. This mirrors findings in a PPS study in South Africa (only 5.6% of prescriptions had no indication recorded)^68^ and is better than the African countries in the GPPSS study where compliance was only 70.4%.^28^ There appeared to be no missed doses of antibiotics, which is contrary to findings in Botswana.^21^ This may be due to the high number of nursing staff in each ward and their level of responsibility for patient care, particularly in KMH where there are few medical staff.

It was also encouraging to see an automatic stop for IV antibiotics at 48 h following high use of IV administration initially (Table 2), with review and represcribing if required. This is seen as a valuable initiative in both hospitals as this limits the unnecessary prolongation of IV therapy and consequently should reduce the risk of IV-related issues, such as phlebitis, and free up nursing time as well as limiting costs. Practice in these hospitals appears to contrast with a lack of review dates and IV-to-oral switching in other sub-Saharan African countries^28^^,^^68^ and may serve as an exemplar to other hospitals in Ghana and more widely within Africa.

Another positive observation in both hospitals was that the duration of oral antibiotics was documented on the medicine chart and usually also in the patient notes in both hospitals. However, the duration of oral therapy was usually 1 week, irrespective of prior IV therapy or indication. This is a concern as there is growing evidence for most acute bacterial infections that ‘shorter is better’ due to lower rates of adverse events including AMR, shorter length of stay and associated reduced costs.^83^ With a standard oral course length of 7 days, even after 2 days of IV treatment, this will be another focus of the education and quality improvement work.

We observed a lack of documentation of any allergies to antibiotics, which was a concern. However, on questioning, it appears that although clinicians do ask patients about allergies these are not routinely recognized or documented. We also did not observe presumptive treatment for infection with MRSA and it is not known if this was because the prevalence of MRSA is low or whether it goes unrecognized through a lack of well-equipped diagnostic facilities, which is commonly observed in LMICs.^17^^,^^84^ Low rates of recognized MRSA infections were recorded in the GPPSS study among the participating centres in Africa (1.2%) versus rates of around 10% in Latin America and in Central and West Asia.^28^ Similarly, no patients were being treated for MDR Gram-negative (MDRGN) infections in either hospital. However, it is unknown whether this reflects a low prevalence or a lack of funded diagnostic services and this will be followed up in future studies.

Compliance with the NSTGs was approximately 65.0% in GPH and 55.0% in KMH, which is similar to other African countries, but falls short of high levels (95%) established in some countries.^39^ This is a key area for improvement given the association with adherence to national guidance and improved patient outcomes, reduced harms and reduced length of stay.^85^ There were many antibiotic prescriptions for indications for which no guidance was available in the NSTGs. Guidance on the prevention of SSIs is not included for many specialities within the NSTGs and the PPS data from both hospitals showed that antibiotics were given for >1 day in approximately 70% of cases and often prolonged for up to 7 days. This is similar to findings in Botswana where the majority of patients in a leading tertiary hospital were given antibiotics for a mean of 5 days post-operatively^74^ and in a study of neurotrauma patients in Kenya,^75^ as well as in the GPPSS study where prolonged surgical prophylaxis (>1 day) was reported in up to 86.3% of cases.^28^ Current guidance advocates that antibiotic prophylaxis should typically be given 1–2 h before surgical incision, with limited further doses^86–89^ as longer duration potentially increases AMR, side-effects and costs with no significant reduction in SSI rates.^28^^,^^74^ Current practice may reflect a lack of national or local guidance but also clinician concerns regarding the risk of infection in a resource-poor setting following surgery. However, studies from African countries have shown the benefits of moving to single-dose prophylaxis without unintended consequences^90^ and this will be followed up in both hospitals.

### Conclusions

This study has established baseline data in these two hospitals to support education and establishment of ASPs to improve future antibiotic utilization. The Global PPS study proved straightforward to undertake with support from experienced partners and there was good engagement from hospital staff. This is important in resource-limited settings, given some of the concerns seen in Botswana and South Africa. Compliance with prescribing guidance could be improved but equally important is to identify and rectify gaps in NSTGs. A number of opportunities for improvement were identified including developing prescribing guidance to suit local needs, i.e. covering the common indications and promoting shorter courses based on current evidence. While IV therapy was commonly used, the duration was often short, which supports early hospital discharge. Development of surgical prophylaxis guidance to reflect WHO recommendations while also addressing local concerns amongst clinicians using a behaviour-change approach should be a priority. There is also an urgent need to improve diagnostics to reliably inform empirical antibiotic choices. We will be following these up as part of ongoing programmes to support ASPs in these two hospitals. A follow-up PPS is planned for 2020 to evaluate the impact of education and quality improvement interventions.

## Supplementary Material

dlaa001_Supplementary_Data
