# Supplementary material for: Antimicrobial point prevalence surveys in two Ghanaian hospitals: opportunities for antimicrobial stewardship
Source: JAC Antimicrob Resist. 2020 Feb 18;2(1):dlaa001. doi: 10.1093/jacamr/dlaa001 (PMC8210261; doi:10.1093/jacamr/dlaa001)
Supplement: dlaa001_Supplementary_Data [file dlaa001_supplementary_data.docx]

**Reviewer report 1 (R0 – review of original submission)**

**PLEASE NOTE: JAC-AMR aims to publish your anonymous peer review comments with the final article for transparency. Please indicate in the box below whether you give your consent for us to publish your peer review comments for this article.**

Yes

**Synopsis**

**1. If the article contains original data, does the synopsis adequately reflect the manuscript as a whole, with the most important aspects described (of the introduction, methods and key results, and a balanced conclusion)? If NO, what is required to improve the synopsis? Please use the box below.**

Yes

Good overall abstract that sets out what the study did and what the findings where. It would benefit somewhere, ideally the conclusion section, from explicitly stating what the novelty of this work is.

I thought it would be useful to highlight (line 43) that the IV duration prescribed was generally short, and there was good oral switching. I would remove "(apart from one patient)" from line 44 as it doesn't add anything.

**Introduction**

**2. Does the introduction provide the necessary background information for readers to understand the article? (e.g. does it describe the problem, current state of knowledge, etc.) If NO, what is required to improve it? Please use the box below**

No

**3. Is the research hypothesis and/or aims/objectives of the study clearly stated in the introduction? If NO, what is required to improve it? Please use the box below**

Yes

**4. Is there superfluous/unnecessary text in the introduction? (Only text that is required for readers to understand why the study has been done should be included). If YES, what text is superfluous and should be removed? Please use the box below**

No

Considering the answers above and any other comments/advice you may have, please indicate what is required to improve the Introduction:

I felt that the introduction was generally lacking some detail that directly related to the context of hospital care, AMS and AMR in countries similar to Ghana. For example:

- "due to variable laboratory resources" on line 69 could be supported by some more discussion on what this variability is, and how it could impact on AMS

- Between lines 69-76 there are around twenty references, grouped together, to support general points. I believe this part of the introduction would benefit from more specific detail around findings and activities in countries similar to Ghana. This level of detail would also be of interest for readers and would help set the scene for the discussion section of this paper.

- WHO AWaRe is also mentioned in the discussion section, which could be introduced earlier in the paper.

- An overview of the findings from the other Ghanaian studies highlighted in lines 84-86 would also be useful in setting the scene for this paper.


Personally, I found the introduction to be a little heavily references in some parts (e.g. lines 60, 72, and 92) for the general points that were being made. The authors should endeavor to align the references closer to any specific points they are making (e.g. split these up and place next to specific items in lists) and focus on the most relevant evidence to support the points made.

**Methods**

**5. Were the methods used suitably robust and appropriate for the research question(s) and/or the aims/objectives of the study? If NO, the article should be rejected, but please advise the authors accordingly in the free-text box below and complete the review in full**

Yes

**6. Are the methods described in enough detail so the reader can understand what was done and interpret the results and conclusions appropriately?**

Yes

**7. Are the methods described in language that a reader who does not specialize in the area of the study/methods used will understand? For example, are the statistical approaches used described in language that non-statistician healthcare professionals will understand? If NO, please indicate what is required to improve the language in the methods section in the box below**

No

Considering the above and any other comments/advice you may have, please indicate other aspects of the methods section that could be improved in the free-text box below)

Overall the methods gave enough detail and sign-posted where necessary. In consideration of a wider readership I would commend the following changes:

- On line 126, give the names of the drugs/classes the ATC codes relate to. While this is referenced, for ease of reading and understanding the methods, and appreciating the level of detail audited, it would be prudent to give drug/class names here.

- On line 127, what qualifies as "short stay patients"? Please give some more detail on this.

- On lines 154-155, the countries compared to should be listed explicitly rather than just giving the references. If the authors do not want to list the countries here, they could refer to a table or figure that includes all the countries. Such a table or figure is not in the current manuscript, but could be depending on how the authors consider the feedback on the results section).

**Results**

**8. Given the methods used, are results described accurately, in enough detail and in an appropriate format to adequately inform the discussion section and the authors/readers conclusions? If NO, please indicate what is required to improve the results section in the box below**

No

**9. Based on what is described in the methods section, are data/results (that are likely to be available to the authors) missing from the current draft that you think should be presented to improve the manuscript?**

No

**10. Are Tables/Figures clear, required and do they add/complement the results text (they should not simply repeat what is in the text or vice versa)? If NO, please indicate what is required to improve the Tables/Figures in the box below**

Yes

Considering the above and any other comments/advice you may have, please indicate what is required to improve the results section:

Overall there was a good level of detail in the results and it was split into subsections for ease of reading, with good supporting figures and tables.

One recommended change that could be made to improve the results and compliment the discussion (relates to the third aim of this study, which is to compare with other hospitals) would be to present figures that show the percentage compliance with certain PPS indicators from the two Ghanaian hospitals, compared to the reference hospitals. While these numbers are given in the discussion, figures that clearly show where these two hospitals sit/rank in relation to comparator hospitals could be visually interesting and be a foundation for the discussion section.


I also had some other questions while reading the results, which should be addressed in the methods, results, or discussion section

- Line 195: Why were neonates excluded from this data?

- Line 204: URTI, ENT, and Bron should be defined in the main text, or the full names given if not used elsewhere in the main text. This should be done irrespective of these being used and defined in table 3.

- Line 214: Why was there a lack of culture and sensitivity testing. Is this due to lack of samples being taken, or due to lack of laboratory resources to process and report?

**Discussion**

**11. Are the discussion and any conclusion(s) reasonable given the stated hypothesis/aims/objectives and results? If NO, please indicate how to improve the discussion in the box below**

Yes

**12. Is there superfluous/unnecessary text in the discussion? (Only text that directly relates to the results and understanding thereof in the context of existing published literature and the limitations of the study should be included.) If YES, please indicate what should be removed to improve the discussion in the box below**

Yes

**13. Is the Limitations sub-section adequate and reflective of the actual limitations of the study? ? If NO, please indicate how to improve the Limitations section in the box below**

No

Considering the above and any other comments/advice you may have, please indicate how to improve the discussion section:

Overall I found the discussion very interesting, particularly seeing how the Ghanaian hospitals audited compared to other PPS studies in similar countries.

There is a major omission in that there is no specific discussion of the limitations of this work. While a lack of HIV, TB, MRSA and MDR patients was mentioned, which could limit the generalisability of the study, I felt that more explicit discussions of the limitations were needed - particularly the well known limitations of PPS studies (e.g. doesn't show trends, doesn't tell you why something is happening, dependent on the staff that day, etc).


Limitations aside, the authors should also consider the following points, which could improve the discussion section:

- Lines 221-227: Could be useful to put the total time and investment required to undertake the PPS, and compare these totals to the literature.

- Line 227 and 229: Pakistan and UK is compared against, although the introduction made me believe the focus was on Africa or other LMICs. If you are going to compare to non-african countries and non-LMICs then this should be clearer earlier in the manuscript. I also feel the UK PPS requires a reference.

- Lines 235-240: As suggested in the results section, I feel this discussion and comparison with other hospitals/countries would benefit from clear figures that show the numbers, possibly in rank order.

- Line 246: "evidenced" would benefit from a reference to back this up.

- WHO AWaRe categories are mentioned around lines 249-254. The discussion may benefit from some debate around the importance and relevance of these to the results and Ghanaian AMS.

- Line 260: States the duration on the medicine chart. Line 149 states in the patients note. Please review and make sure these are clear and aligned (was it notes, chart, or both?).

- Lines 263-265: Is there any explanation or discussion around why there were no missed doses of antimicrobials, compared to the literature?

- Lines 268-269: "IV device-related harm" would benefit from a reference. I assume this relates to IV catheter infections, so the authors could also consider other IV related issues such as drug-administration errors, nursing time (quicker to give PO than IV), and costs (generally more expensive than oral forms).

- Line 272: Looks like there is a superfluous "c" at the end of this paragraph.

- Lines 273-278: I feel the "shorter is better" discussion point would benefit from expansion. Why is it better (e.g. ADRs, AMR, Costs, etc)? What is the average oversupply/prescribing seen? (e.g. median 1 day, or 3 days)?

- Line 289: review spelling of "serviceses" (7th word on line).

- Line 293: "Quality of antibiotic prescribing". What does this mean for patients, services, and AMR? There is evidence to show that abiding to treatment guidelines improves patient outcomes, reduces harms, and reduces length of stay. This is a much more important reason for following guidelines.

- Line 289 and 299: This discussion point would benefit from some specific numbers on how many peoples received the stated durations of antimicrobials, as  this has been given for the global PPS on line 301.

- Line 303 and 304: Suggest changing "without impacting on SSI rates" to "with no significant reduction in SSI rates" as there is some data that longer prophylaxis for some procedures does reduce SSI and other infection rates. However, this small benefit isn't felt to outweigh the risks from the drugs and also AMR.

**General**

**14. Is referencing throughout the manuscript satisfactory? (for instance are there statements for which you believe a supporting reference should be cited?) If NO, please indicate how to improve referencing in the box below**

No

**15. Is the language used (i.e. spelling, grammar, etc.) of high enough quality to be published as is? If NO, please indicate how to improve the language within the article in the box below**

No

**16. Are ethics/other approvals appropriate/adequate and satisfactorily described? ? If NO, please indicate how to improve this in the box below**

Yes

Considering the above and any other comments/advice you may have, please advise the authors how to improve these aspects of the article.

Referencing, spelling and grammar has been discussed above.

**Opinion and review articles only**

**17. Please advise the authors in the box below on how to improve their opinion or review article:**

n/a

|  **Recommendation** | |
| --- | --- |
|  | Accept (as is with no revisions) |
|  | Minor revision (with anticipation of subsequent acceptance if revisions are adequate) |
|  | Major Revision |
|  | Reject |

| **Would you be willing to review a revision of this manuscript?** | |
| --- | --- |
|  | Yes |
|  | No |

**Comments**

 Comments to the Author

If you have any additional comments to the authors about any aspect of the manuscript not covered in the structured review above, please place these in the box below. If you have no additional comments, please say “No additional comments” in box.

Overall I enjoyed reading this manuscript and found it very interesting. This is extremely important work and the collaborative approach to improving antimicrobial prescribing should be commended - and will lend itself to further improvement work and dissemination among similar healthcare settings. The specific comments made are aimed to help improve the manuscript to make it easier to read and apply in those other settings, where context is important to improving AMS.
